# MiR-205-3p suppresses bladder cancer progression via GLO1 mediated P38/ERK activation

**DOI:** 10.1186/s12885-023-11175-9

**Published:** 2023-10-09

**Authors:** Zou Zhenhai, Cheng Qi, Zhang Shuchao, Wang Zhongqi, Song Xue, Geng Zhijun, Mei Zhijie, Liu Jianmin, Liu Beibei, Guo Yuanyuan

**Affiliations:** 1 Department of Urology, The First Affiliated Hospital of Bengbu Medical College, No.287, Changhuai Road, 233040 Anhui, Bengbu, Longzihu, Bengbu China; 2 The Central Laboratory of the First Affiliated Hospital of Bengbu Medical College, Anhui, Bengbu, 233040 China

**Keywords:** Bladder cancer, MiR-205-3p, GLO1, P38/ERK

## Abstract

MicroRNAs (miRNAs) have been reported to serve as potential biomarkers in bladder cancer and play important roles in cancer progression. This study aimed to investigate the biological role of miR-205-3p in bladder cancer. We showed that miR-205-3p was significantly down-regulated in bladder cancer tissues and cells. Moreover, overexpression of miR-205-3p inhibited bladder cancer progression in vitro. Then we confirmed that GLO1, a downstream target of miR-205-3p, mediated the effect of miR-205-3p on bladder cancer cells. In addition, we found that miR-205-3p inhibits P38/ERK activation through repressing GLO1. Eventually, we confirmed that miR-205-3p inhibits the occurrence and progress of bladder cancer by targeting GLO1 in vivo by nude mouse tumorigenesis and immunohistochemistry. In a word, miR-205-3p inhibits proliferation and metastasis of bladder cancer cells by activating the GLO1 mediated P38/ERK signaling pathway and that may be a potential therapeutic target for bladder cancer.

**Supplementary Information:**

The online version contains supplementary material available at 10.1186/s12885-023-11175-9.

## Introduction

Bladder cancer is the most common malignant tumor of urogenital system, of which more than 90% is urothelial cancer, which has biological characteristics such as multicentric origin, easy recurrence, metastasis and drug resistance. In 2020, there were 573,278 new cases of BCa and 212,536 deaths worldwide. Smoking, genetic factors and exposure to aromatic amines are considered to be the main causes of bladder cancer [1, 2]. Approximately 70% of bladder cancers are non-muscle invasive bladder cancer [3, 4], and these patients often require multiple transurethral cystectomies, high-frequency bladder infusion chemotherapy, and intensive cystoscopic follow-up, but even so, 30-45% of patients progress to muscle-invasive or metastatic bladder cancer within 5 years [5]. Therefore, it is important to investigate the biological mechanisms of bladder cancer development and progression.

miRNAs are small single-stranded RNAs (18–25 nucleotides) that function as post-transcriptional repressors primarily by binding to complementary sequences in the 3´-untranslated region of their target mRNAs [6]. However, many miRNAs in bladder cancer have not been fully explored, their functions and target genes need to be further elucidated. MiR-205-3p is relevant to the development of many tumors and has changed expression levels in different tumors, such as low expression in gastric [7] and ovarian cancers [8] but high expression in non-small cell lung cancer [9], breast carcinoma [10] and other tumors. However, there are no any studies have reported the effect of miR-205-3p on the biological behavior of bladder cancer and the potential mechanisms are still unknown.

Glyoxalase 1 (GLO1) is a key cytoprotective enzyme associated with the degradation function of methylglyoxal (MG) [11]. Abnormal GLO1 expression leads to the production of advanced glycosylation end products (AGEs) by dienyl stress, and the accumulation of AGE-modified proteins causes age-related diseases, such as obesity, cardiovascular disease and diabetes [12]. High expression of GLO1 has been observed in common tumors, including lung, liver, breast, prostate cancers, and so on [13–16], and that is associated closely to some biological functions such as cells proliferation, apoptosis and metastasis.

In present study, we observed the role of miR-205-3p in bladder cancer progression, and further identified that miR-205-3p inhibited bladder cancer by targeting GLO1. Moreover, we found P38/ERK signaling pathway participated in the procedure of GLO1 influencing bladder cancer. The study provides a potential therapeutic target and theoretical basis for the clinical treatment of bladder cancer.

## Methods

### Cell lines and culture

The normal human uroepithelial cell line SV-HUC-1, and the bladder cancer cell lines T24, 5637 and EJ were obtained from the Shanghai Cell Bank of the Chinese Academy of Sciences.Normal human uroepithelial cell line SV-HUC-1 was cultured in F-12 K medium containing 10% fetal bovine serumand and bladder cancer cell lines T24, 5637 and EJ cells were cultured in RPMI-1640 medium containing 10% fetal bovine serum. The cells were digested and passaged with 0.25% trypsin when the cell fusion was around 80%, with fluid changes or passages every 2 days.RPMI-1640 medium, F-12 K medium,fetal bovine serum, penicillin-streptomycin antibody and trypsin were purchased from Gibco Company of the United States.

### Bioinformatics analysis

Downloaded the differentially expressed miRNA dataset associated with BLCA from the TCGA database (https://www.cancer.gov/tcga) and used the edgeR package in R software (v3.5.2) to calculate the differentially expressed miRNAs with the screening conditions logFoldChange = 1, padj = 0.05. Volcano and heatmap maps were plotted using the plot function and pheatmap package. Then analyzed the relationship between miR-205-3p expression and survival of BLCA patients using the KM plotter website (http://kmplot.com/analysis/) and plotted the corresponding survival curves.

### Clinical specimen

Bladder cancer tissue and paracancerous tissue were obtained from 35 bladder cancer patients in the First Affiliated Hospital of Bengbu Medical College from December 2018 to December 2021. None of them received preoperative radiotherapy or preoperative chemotherapy. All specimens were processed according to ethical and legal standards. This study was approved by the Ethics Committee of the First Affiliated Hospital of Bengbu Medical College [2022] No. 144.

### Lentiviral vector construction and cell transfection

The lentivirus containing the miR-205-3p overexpression construct was produced by Genomeditech (Shanghai, China). Cells were transfected according to the manufacturer’s protocol. The efficiency of gene overexpression was verified by quantitative reverse transcription polymerase chain reaction (RT-qPCR) assay.

### Reverse transcription-quantitative polymerase chain reaction (RT-qPCR)

Total RNA was isolated from tissues and cells using Trizol reagent and reverse transcribed into complementary deoxyribonucleic acid (cDNA). After pre-denaturation at 95 °C for 1 min, denaturation at 95 °C for 20 s and annealing at 60 °C for 20 s, a total of 40 cycles, the 2^(-ΔΔCt) method was used to calculate the relative expression of the target genes. Primers were designed using Primer 5. 0 (Table 1).

**Table 1:** primer sequence

Gene	primer sequence(5’-3’)
miR-205-3p-F	CGCGCTGTACAGGCCACTG
U6-F	CGCTTCGGCAGCACATATACTAA
Micro-R	AGTGCAGGGTCCGAGGTATT
GLO1-F	CGGGGCAAAATGTCCTCGTA
GLO1-R	CGGAAGAGTCCGGGTGTTT
GAPDH-F	GGAGCGAGATCCCTCCAAAAT
GAPDH-R	GGCTGTTGTCATACTTCTCATGG

### Cell counting kit-8 assay

Collection of cells after 24 h of transfection,cells were inoculated in 96-well plates with 2 × 10^3 cells/plate and cultured for 24 h, 48 and 72 h, respectively. 10µL CCK8 solution was added to each well, protected from light and cultured for 1 h at 37℃. The absorbance value of the cells at 450 nm (OD450nm value) was detected by enzyme marker and the experiment was repeated 3 times.

### Transwell assay

In the cell invasion assay, Matrigel gel was prepared on the upper chamber surface.Collection of cells after 24 h of transfection,,cells were adjusted to 2 × 10^4 cells/ml with serum-free medium and 100µL was aspirated and added to the upper chamber. In the lower chamber, 1640 medium containing 20% fetal bovine serum was added. 24 h of incubation at 37 °C, the cells and Matrigel gel were gently removed from the upper chamber, fixed in 4% paraformaldehyde for 30 min and then stained with crystalline violet, photographed and analyzed under a microscope. For the cell migration assay, no Matrigel gel was added to the upper chamber of the Transwell and the rest of the steps were the same as for the cell invasion assay, which was repeated three times.

### Flow cytometry

Collection of cells after 24 h of transfection, take 50,000-100,000 resuspended cells, centrifuge at 1000 g for 5 min and discard the supernatant. 195µLAnnexin V-FITC conjugate was added to resuspend the cells. Add 5µL Annexin V-FITC, 10µL of propidium iodide staining solution, mix well and incubate for 10-20 min at room temperature, flow-on assay, and repeat the experiment 3 times.

### Transcriptome sequencing

Three samples of each group were collected from cells after 24 h of transfection, and total RNA was extracted using TRIzol reagent. The purity, concentration and integrity of the RNA samples were checked by Nanodrop spectrophotometer (ND-ONE-W) for library construction. mRNA was randomly interrupted by adding Fragmentation Buffer, and the mRNA was used as a template to synthesize the first cDNA strand with six-base random the first cDNA strand was synthesized using random hexamers as template, and the second cDNA strand was synthesized by adding buffer, dNTPs, RNase H and DNA polymerase I. The cDNA was purified using AMPure XP magnetic beads, and the purified double-stranded cDNA was then end-repaired, A-tailed and connected to sequencing junctions. The purified cDNA was then end-repaired, A-tailed and sequenced, followed by fragment size selection using AMPure XP beads, and finally enriched by PCR to obtain a cDNA library. After library construction, the effective library concentration (effective library concentration > 2nM) was accurately quantified using the RT-qPCR method to ensure library quality. After passing the library check, the different libraries were mixed according to the target downstream data volume and sequenced using the Illumina platform.

### Dual-luciferase reporter assay

Construction of GLO1 wild-type and mutant plasmids, the tool cells were inoculated in 6-well plates and transfected with GLO1 wild-type plasmid, GLO1 mutant plasmid, mimics-NC and mir-205-3p mimics, respectively or simultaneously, and the luciferase activity of each group was measured using a dual luciferase reporter gene kit.

### Western blot assay

Cells or tissues were collected and washed twice with cold PBS, lysed on ice for 30 min after the addition of RIPA lysate, the lysates were centrifuged at 4 °C, 12,000 r/min for 15 min and the protein concentration in the supernatant was determined using the BCA protein quantification kit. Equal amounts of protein samples were added to SDS-PAGE and transferred to PVDF membranes, followed by closure with 5% skimmed milk powder for 1 h and incubation of the primary antibody on a shaker at 4 °C overnight. On day 2 secondary antibodies were incubated at room temperature for 1 h, washed 3 times with TBST and protein bands were observed using chemiluminescent reagents and analyzed using Image J.

### Xenograft mouse model

Ten BALB/c nude mice were fed at 25℃and 60–70% humidity. The constructed lentivirus was transfected into the tumor cells and after confirming successful transfection, the cell suspension was injected subcutaneously into the nude mice at a density of 4 × 10. After 28 days, the nude mice were executed and the tumor volume was measured and weighed using vernier calipers and the tumor was used for the next immunohistochemistry. All experimental operations were performed in accordance with the relevant regulations of the NIH Guide for the Use of Laboratory Animals. The experimental protocol was approved by Institutional Animal Care & Use Committee of the First Affiliated Hospital of Bengbu Medical College.

### Immunohistochemistry (IHC)

Tissue specimens were paraffin-embedded and sectioned, immunohistochemistry was performed according to the kit, diaminobenzidine was used for color development, hematoxylin was used for re-staining and xylene was used to seal the sections. The IHC assessment score for each sample was based on the product of the staining intensity score and the range of stained areas.The antibodies used are Ki-67 (D3B5) Rabbit mAb and GLO1 (GLO1a) Rabbit mAb.

### Statistical analysis

Gene expression data were log-transformed and normalised for statistical analysis, while graphs were constructed using R software 3.6.2 and the Perl language package. SPSS 22.0 statistical software and GraphPad Prism9 were used to analyse and process the experimental data. Measures were expressed as x ± s and t-tests and one-way ANOVA were used to assess statistical differences, with differences considered statistically significant at P < 0.05.

## Results

### MiR-205-3p is down-regulated in bladder cancer and negatively associated with clinical prognosis

**Fig. 1:**
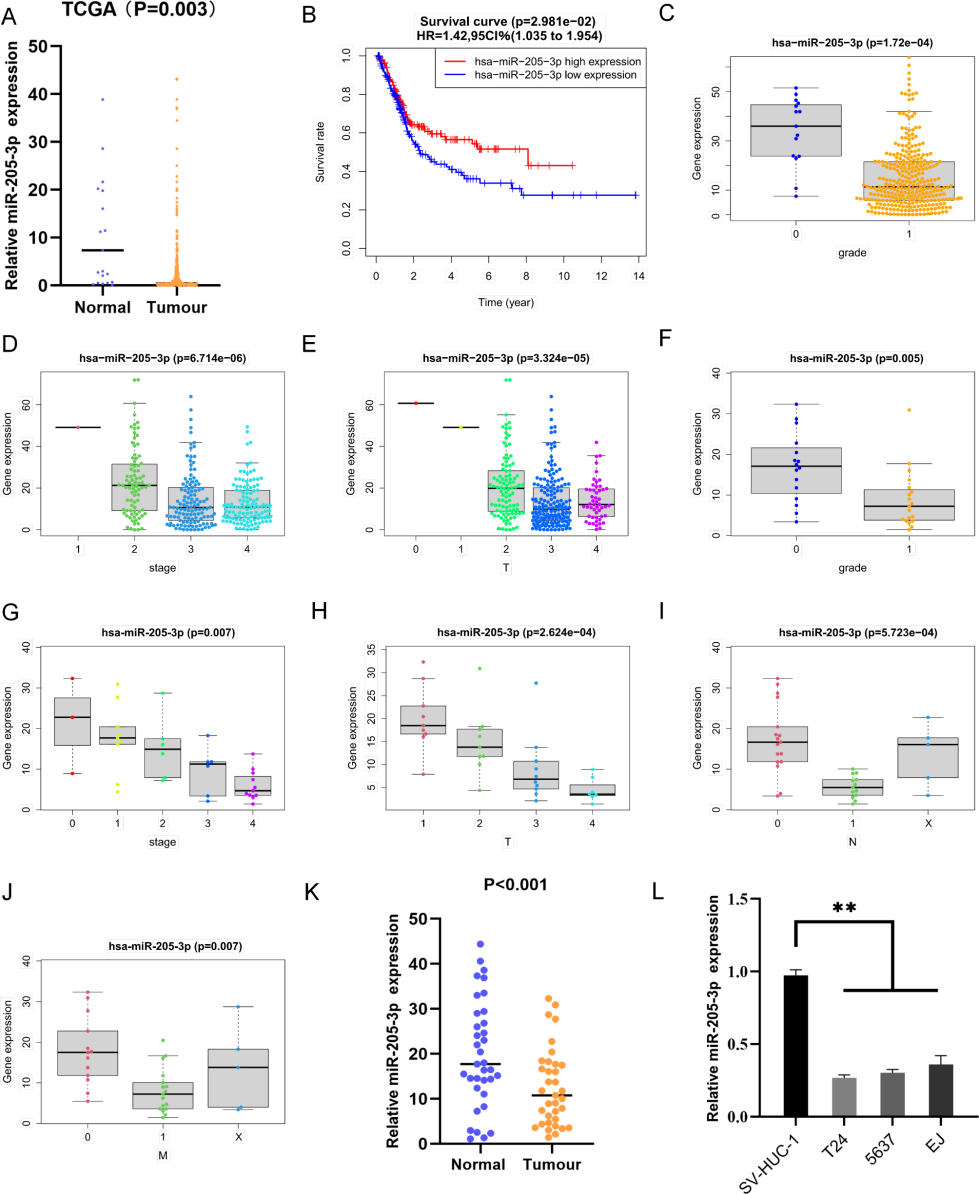
Decreased miR-205-3p expression is associated with poor prognosis of patients with bladder cancer. **(A)** The expression of miR-205-3p in 418 bladder cancer tissues and 19 normal tissues from TCGA dataset. **(B)** The KM-plotter curve between high expression group and low expression group of mir-205-3p. **(C, D and E)** The association between miR-205-3p expression and the clinical grade, stage and T-stage of bladder cancer patients. **(F)** MiR-205-3p mRNA expressions in normal tissues and bladder cancer tissues. **(G)** MiR-205-3p mRNA expression in bladder cancer cell lines T24, 5637, EJ and normal SV-HUC-1 cells

To investigate the expression level and clinical significance of miR-205-3p in bladder cancer, miRNA data of patients, including 418 bladder cancers and 19 normal individuals, were downloaded and analyzed based on the TCGA database. The results showed that the expression of miR-205-3p was sharply down-regulated in bladder cancer tissues compared with that in normal tissues (Fig. 1A). The patients with low miR-205-3p expression exhibited shorter median OS of 11.5 months than the patients with high miR-205-3p, whose median OS were 16.3 months (Fig. 1B). In addition, The relationship between clinicopathological characteristics and miR-205-3p was analysed using Wilcoxon signed rank test and logistic regression (Fig. 1C, D and E). Then we detected miR-205-3p expressions in bladder cancer tissues and normal bladder tissues by qRT-PCR, the result was consistent with that from the TCGA database (Fig. 1K),The clinicopathological data of bladder cancer patients were also statistically analysed accordingly with miR-205-3p expression. The higher the expression of miR-205-3p, the lower the pathological grade of the patients(Fig. 1F-J). Moreover, the expressions of miR-205-3p were also decreased in three bladder cancer cell lines (T24, 5637, EJ), compared to normal uroepithelial cells SV-HUC-1 (Fig. 1L). Therefore, we speculate that reduced miR-205-3p expression is closely related to the development of bladder cancer.

### MiR-205-3p inhibits proliferation, migration, invasion and induces apoptosis in bladder cancer cells

**Fig. 2:**
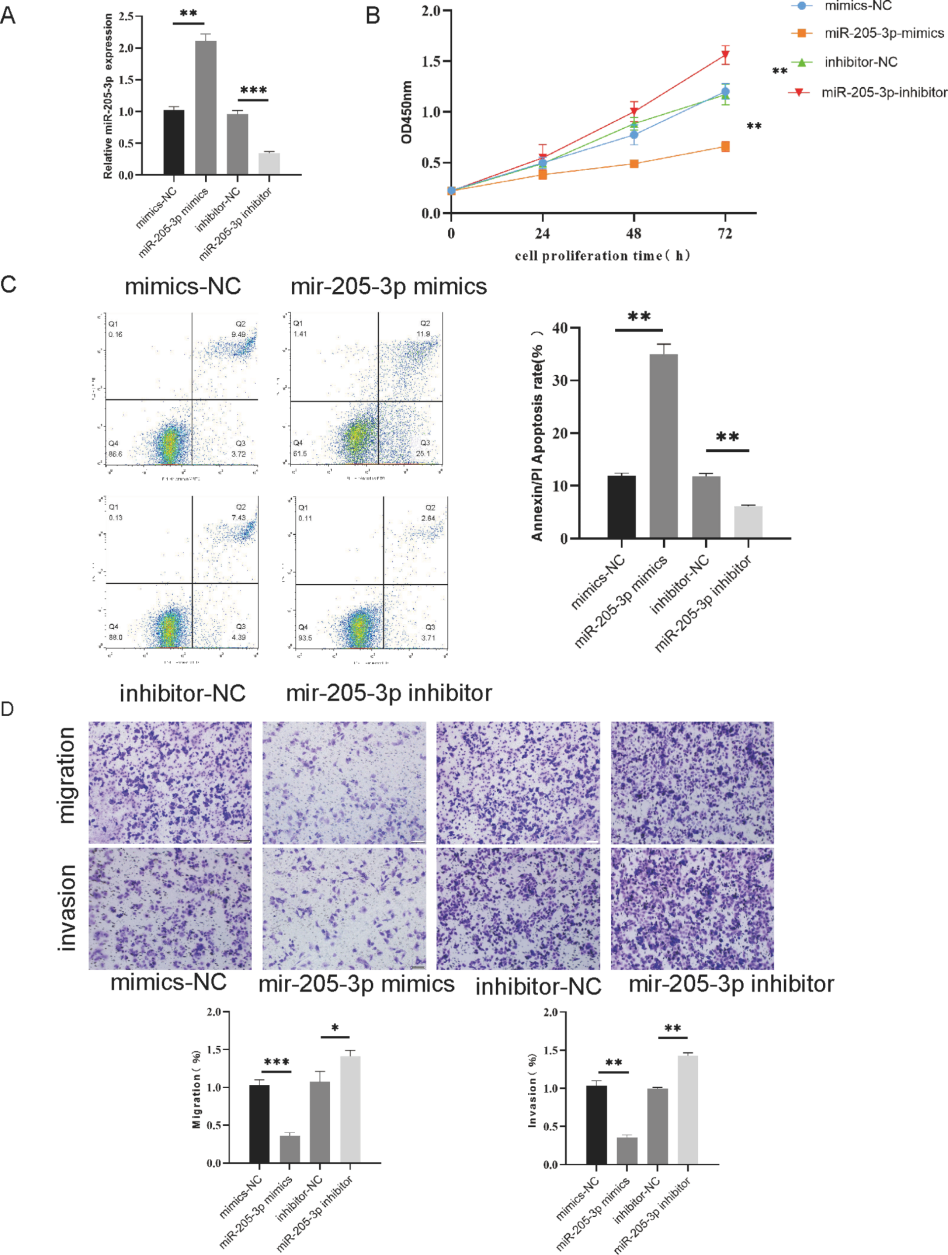
MiR-205-3p inhibits proliferation, migration, invasion and induces apoptosis in bladder cancer cells. **(A)** MiR-205-3p expressions were determined by RT-qPCR in T24 cells with miR-205-3p knock-down or overexpression. **(B)** The cell proliferation abilities were assessed by CCK8. **(C)** The cell apoptosis was detected by flow cytometric analysis (Annexin Fitc/PI). **(D)** The capacities of migratory and invasion of T24 cells was detected by Transwell assays

To explore the role of miR-205-3p in bladder cancer progression, miR-205-3p was up-regulated and down-regulated respectively by transfecting according plasmids into T24 cells, the transfection efficiency was verified by RT-qPCR (Fig. 2A). CCK8 assay showed that the proliferation capacity was significantly reduced in the miR-205-3p mimics group than that in the mimics-NC group, and the proliferation ability of miR-205-3p inhibitor group was higher than that of inhibitor-NC group(Fig. 2B). Accordingly, the migration and invasion capacities were decreased when miR-205-3p was up-regulated (Fig. 2D). In addition, flow cytometry analysis showed that apoptosis was significantly increased in the miR-205-3p mimics group and reduced compared with that in the inhibitor-NC group (Fig. 2C). These results suggest that miR-205-3p may inhibit biologic behavior and induce apoptosis in bladder cancer cells.

### MiR-205-3p negatively regulates GLO1

**Fig. 3:**
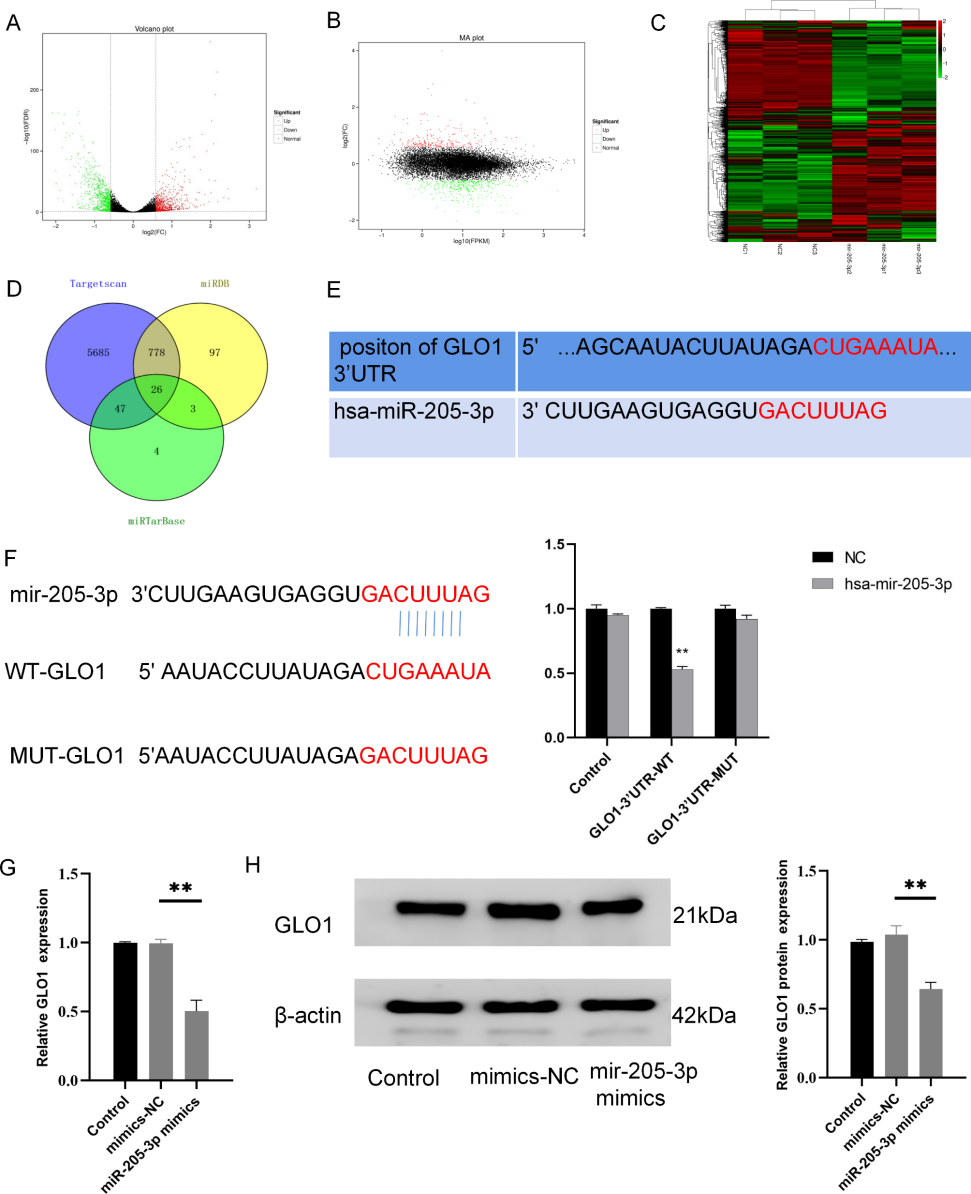
MiR-205-3p regulates GLO1 expression by targeting 3’-UTR binding. **(A)** Volcano plot of transcriptome sequencing. **(B)** MA plot of Transcriptome sequencing. **(C)** The heat map of transcriptome sequencing. **(D)** Wayne chart way of bioinformatics analysis. **(E)** Binding site between miR-205-3p and the 3’-UTR of GLO1. **(F)** The binding relationship between miR-205-3p and GLO1 verified by dual-luciferase assays. **(G)** RT-qPCR was used to detect the expression of GLO1 in cells with miR-205-3p overexpression. **(H)** The protein expressions of GLO1 were observed by western-blot

To identify target genes in the downstream of miR-205-3p, we sequenced the transcriptome in T24 bladder cancer cells over-expressing miR-205-3p (all biological repeat correlations were above 99% for the same group of samples, and a total of 73.38 Gb of total bases were obtained after sequencing quality control), the results showed that the miR-205-3p mimics group had 1597 differentially expressed genes compared to the mimics-NC group, including 718 up-regulated genes and 879 down-regulated genes (Fig. 3A-C). At the same time, three online databases of Targetscan, MiRDB and miRTabase were used for predicting downstream target genes of miR-205-3p, and a total of 26 intersected genes were obtained (Fig. 3D). Among them, however, only GLO1 gene showed down-regulated expression in the sequencing results, and all three online databases also indicated binding sites between miR-205-3p and GLO1 (Fig. 3E). So, we observed whether miR-205-3p regulated GLO1 expression. The results of the dual-luciferase reporter assay showed that the luciferase activity of T24 cells transfected with miR-205-3p mimics was significantly reduced in wild-type GLO1, while in mutant GLO1 there was no significant difference between the two (Fig. 3F), suggesting a binding site between miR-205-3p and GLO1. Moreover, RT-qPCR and western blot showed that both gene and protein expressions of GLO1 were greatly reduced in miR-205-3p over-expressed bladder cancer cells (Fig. 3G H). Taken together, miR-205-3p can negatively regulates GLO1 in bladder cancer cell.

### Mir-205-3p inhibits cell proliferation and metastasis through inhibition of GLO1

**Fig. 4:**
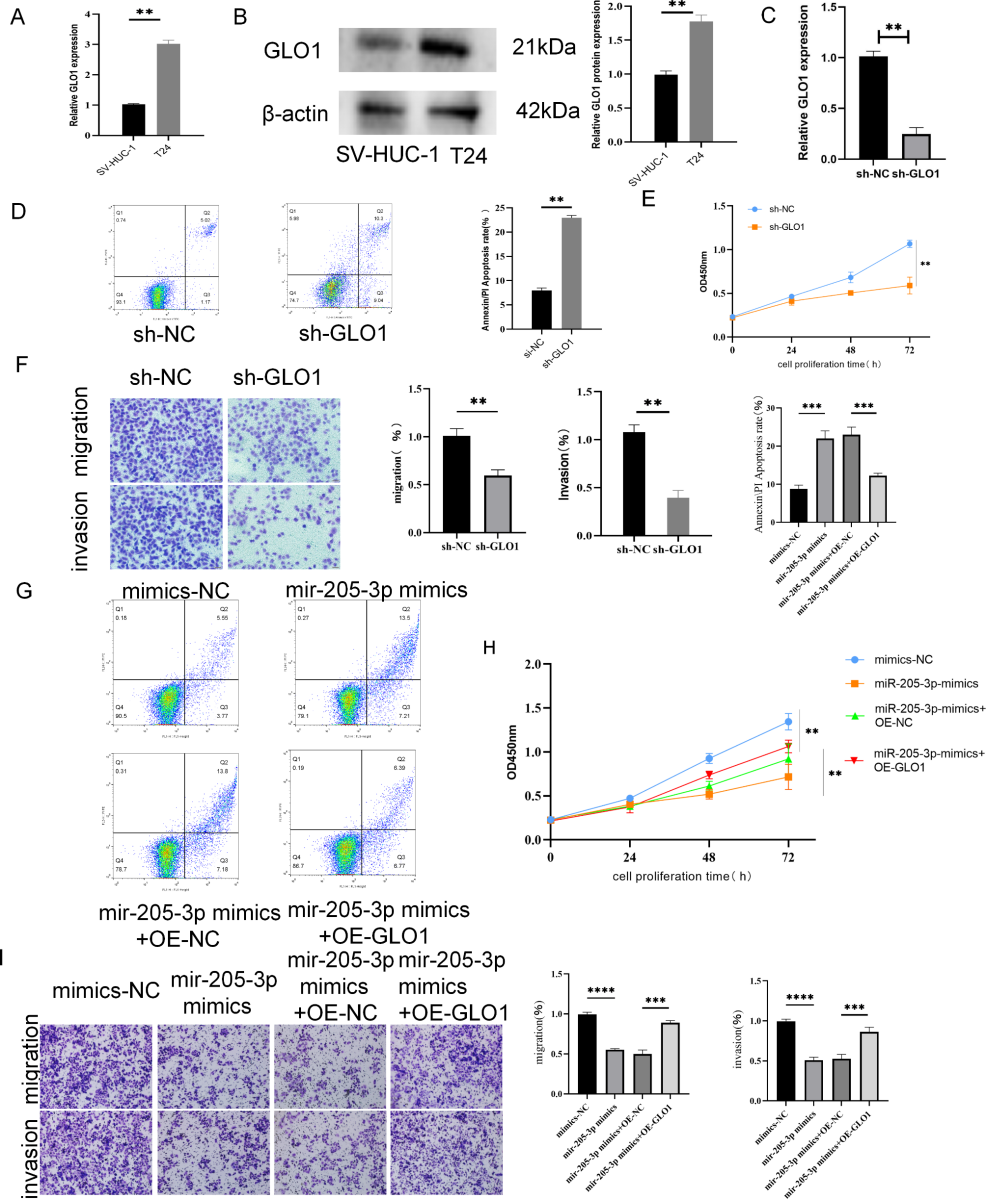
MiR-205-3p inhibits bladder cancer cells by suppressing GLO1 expression. **(A)** The expressions of GLO1 were detected in HUC and T24 cells by RT-qPCR. **(B)** The protein expressions of GLO1 were examined by western-blot. **(C)** The expression of GLO1 was detected by RT-qPCR in cells with knockdown of GLO1. **(D)** The cell apoptosis in T24 cells was detected by flow cytometric analysis (Annexin Fitc/PI). **(E-F)** The ability of proliferation (E), migratory and invasion (F) were analyzed by CCK8 and transwell assays. **(G)** The cell apoptosis was detected by flow cytometric analysis (Annexin Fitc/PI). **(H-I)** The ability of proliferation **(H),** migratory and invasion **(I)** were analyzed in cells with different treatment

To further observed the role of GLO1 in miR-205-3p regulating bladder cancer, we firstly detected the effect of GLO1 expression on bladder cancer cells. As shown in Fig. 4A and B, GLO1 was highly expressed in T24 bladder cancer cells in gene and protein levels. Then, after knocking down GLO1 expression by transfecting shGLO1 (Fig. 4C), flow cytometry, CCK8 and transwell assay were applied to identify the influences of GLO1 on apoptosis, proliferation, migration and invasion. The results demonstrated that the GLO1 down-regulated cells exhibited more apoptotic cells (Fig. 4D) and inhibited proliferation, migration and invasion abilities (Fig. 4E and F), and over-expressed GLO1 showed conversed effects on bladder cancer cells. Moreover, up-regulating GLO1 could reversed the inhibitory effect of miR-205-3p on bladder cancer cells (Fig. 4G and I). These results suggest that miR-205-3p inhibits the proliferation and metastasis and induces apoptosis by suppressing the expression of GLO1.

### MiR-205-3p suppresses bladder cancer progression via GLO1 mediated P38/ERK activation

**Fig. 5:**
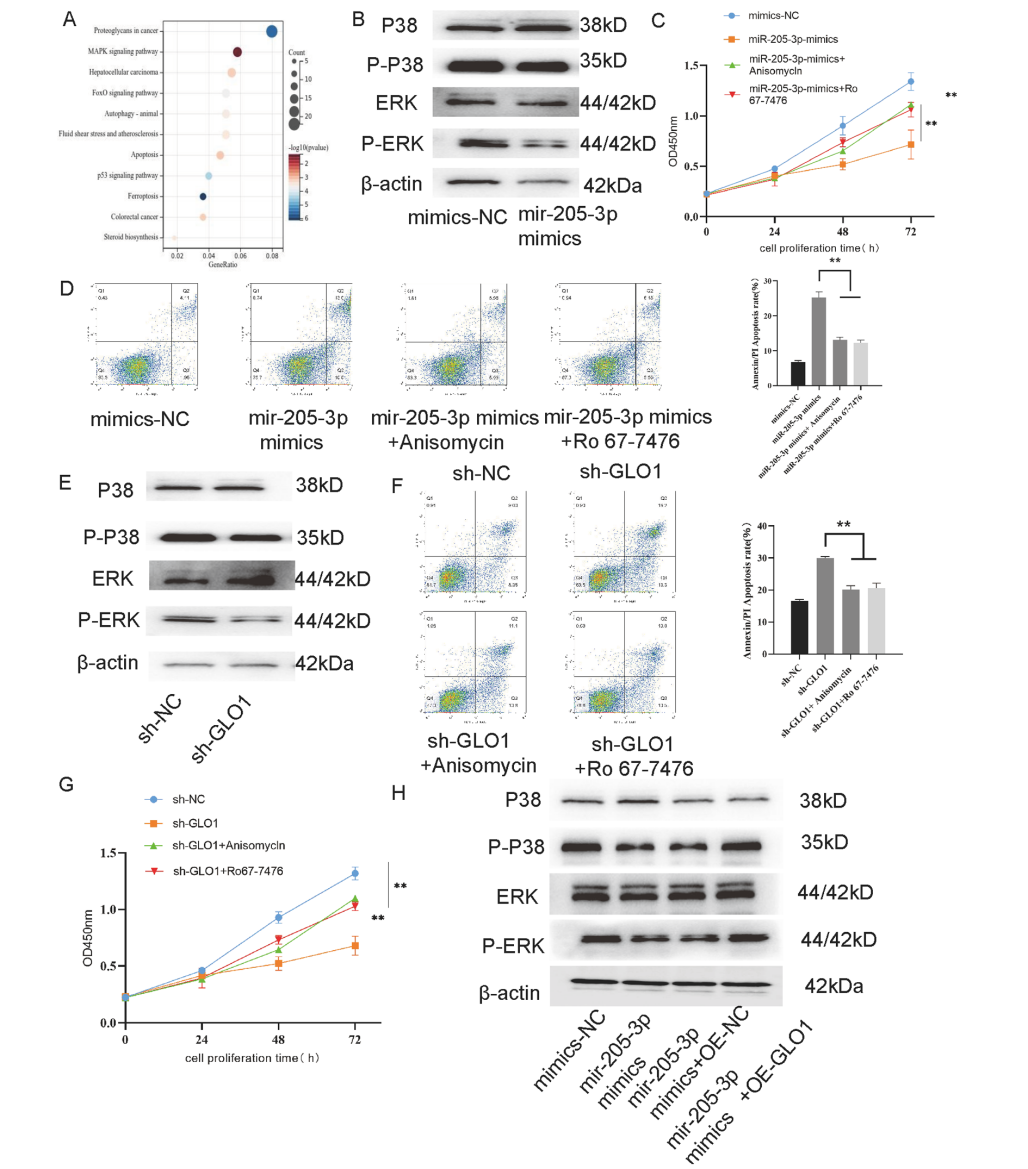
MiR-205-3p via GLO1 regulates the P38/ERK signaling. **(A)** Bubble plots showed KEGG signal enrichment analysis of target gene. Kanehisa,M.and Goto,S.;KEGG:Kyoto Encyclopedia of Genes and Genomes.Nucleic Acids Res.28,17–30(2000). **(B)** the extents of P38 and ERK phosphorylation in T24 cells with miR-205-3p-mimics determined by western blot. **(C)** The effect of Anisomycln and Ro67-7476 in T24 cells with miR-205-3p-mimics cell proliferation ability was assessed by the CCK-8. **(D)** The effect of Anisomycln and Ro67-7476 in T24 cells with miR-205-3p-mimcs cell apoptosis was detected by flow cytometric analysis (Annexin Fitc/PI). **(E)** the extents of P38 and ERK phosphorylation in T24 cells with sh-GLO1 determined by western blot. **(F)** The effect of Anisomycln and Ro67-7476 in T24 cells with sh-GLO1 cell proliferation ability was assessed by the CCK-8. **(G)** The effect of Anisomycln and Ro67-7476 in T24 cells with sh-GLO1 cell apoptosis was detected by flow cytometric analysis (Annexin Fitc/PI). **(H)** the extents of P38 and ERK phosphorylation in T24 cells with miR-205-3p-mimics and sh-GLO1 determined by western blot

To clarify the potential mechanism by which miR-205-3p and GLO1 affect the biological functions of bladder cancer, we performed KEGG pathway enrichment analysis based on the transcriptome sequencing results. Most of differentially expressed genes were associated with proteoglycans, apoptosis and some oncogenic pathways, such as MAPK and P53 signaling pathways in bladder cancer (Fig. 5A). So, we used western blotting to assess the extent of phosphorylation of p38 and ERK. We found that their phosphorylation levels were significantly inhibited in miR-205-3p over-expressed cells (Fig. 5B). To be expected, P38 and ERK agonist reversed the effects of miR-205-3p on bladder cancer cells, both two agonists promoted the cell proliferation and inhibited apoptosis in miR-205-3p up-regulated cells (Fig. 5C and D). On the other hand, GLO1 down-regulation also repressed phosphorylation of p38 and ERK (Fig. 5E) and demonstrated similar effects on cells to the miR-205-3p over-expression, and P38 and ERK agonist were rescued partially these influences, including cell proliferation and apoptosis (Fig. 5F and G). Moreover, western blot results showed that over-expressing GLO1 increased the phosphorylation levels of p38 and ERK, which had been decreased by miR-205-3p (Fig. 5H). All above results reveal that miR-205-3p inhibits P38/ERK activation and cell progression through repressing GLO1.

### MiR-205-3p inhibits tumor growth and GLO1 expression in vivo

**Fig. 6:**
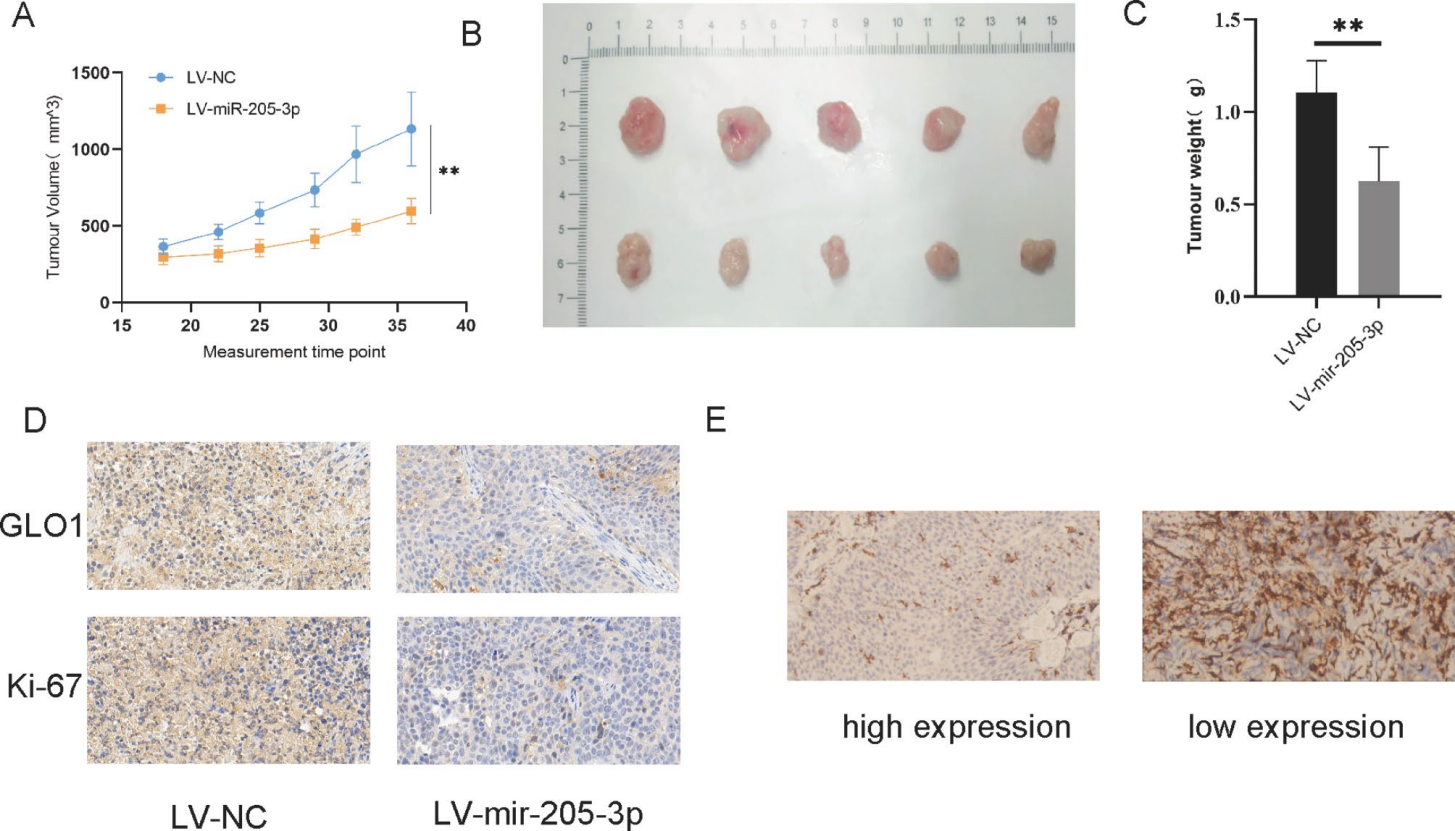
Upregulation of miR-205-3p inhibits BLCA growth and GLO1 expression in vivo. **(A)** Statistical comparison of differences in tumor volume between the LV-NC and LV-miR-205-3p groups. **(B)** Image formation of subcutaneous tumor in nude mice. **(C)** Statistical comparison of differences in tumor weight between he LV-NC and LV-miR-205-3p groups. **(D)** IHC analysis of GLO1 and KI-67 expression levels in xenograft tumor tissue from the two groups. **(E)** IHC analysis of GLO1 expression levels between the high miR-205-3p expression and low miR-205-3p expression groups

To assess the function of miR-205-3p in bladder cancer oncogenesis in vivo, we injected T24 cells stably expressing miR-205-3p as well as the corresponding negative control into nude mice to construct a xenograft model. The results showed that tumor growth was evidently slower (Fig. 6A) the volume was smaller (Fig. 6B) and the tumor weight was more lighter (Fig. 6C) in miR-205-3p over-expression mice than that in control mice. At the same time, IHC assay was performed and confirmed that miR-205-3p increased tumor tissues had stronger protein expression of GLO1 and Ki-67 (Fig. 6D), which hinted that miR-205-3p is related to GLO1 expression and proliferation of bladder cancer. In addition, we also detected GLO1 protein levels in bladder cancer patients with high and low miR-205-3p expression. The results showed that GLO1 protein expression was down-regulated in tumor tissues with high miR-205-3p expression (Fig. 6E). These data suggested again that miR-205-3p inhibits tumor growth and GLO1 expression.

## Discussion

Bladder cancer is the ninth most common malignancy worldwide, with approximately 356,000 new cases and 145,000 deaths per year, has a propensity for recurrence and requires lifelong monitoring after diagnosis [17, 18]. MiRNAs are a class of short non-coding RNAs that bind to complementary sequences on target mRNAs and induce mRNA silencing by inhibiting the translation of proteins or increasing the degradation of gene silencing [19, 20]. This research focused on miR-205-3p and revealed the role of miR-205-3p in regulating bladder cancer cell proliferation, migration, invasion and apoptosis and its associated molecular mechanisms downstream. Firstly, through bioinformatic analysis, we determined that miR-205-3p was lowly expressed in BLCA and that low miR-205-3p expression was usually associated with poorer OS rates in bladder cancer patients. Furthermore, Cox regression analysis showed that miR-205-3p was strongly associated with the patient’s grade, stage and T-stage. Molecular biology experiments also confirmed the low expression of miR-205-3p in bladder cancer tissues and cell lines.All these results suggested that miR-205-3p was low expressed in bladder cancer and inhibited the development of the tumor.

To investigate the role of miR-205-3p in bladder cancer tumorigenesis and progression, we conducted a series of cellular functional assays. Overexpression of miR-205-3p revealed that the proliferation, migration and invasion of bladder cancer cells were inhibited along with increased apoptosis, while down-regulation of miR-205-3p showed the opposite results, suggesting that miR-205-3p could inhibit the biological behavior of bladder cancer cells. MicroRNA-205 was first identified in mouse and pufferfish sequences. In the previous studies, miR-205-3p down-regulated CXCL11 protein and inhibited Akt signalling activation thereby hindering GC cell proliferation and invasion [7], while miR-205-3p was significantly increased in NSCLC tissues by targeting APBB2 to promote NSCLC progression [21], the results demonstrate that miR-205-3p plays a role as a pro-oncogenic factor in NSCLC, possibly due to the opposite effects of miR-205-3p in different tumor cells.

miRNAs typically target one or more mRNAs, causing direct degradation or translational repression of mRNA through specific base pairing with downstream target mRNAs [22], and regulating gene expression at the post-transcriptional level, thereby regulating cell differentiation, growth, proliferation, migration and apoptosis [23, 24]. Therefore, we sequenced the transcriptome of bladder cancer cells overexpressing miR-205-3p, and the sequencing results indicated that GLO1 was significantly downregulated in the miR-205-3p mimics group, in which the expression of GLO1 gene was significantly reduced and the presence of miR-205-3p in the 3’UTR region of GLO1 in three online bioinformatics analyses of sites with the miR-205-3p base binding site. Meanwhile, dual luciferase reporter assays verified the targeting relationship between miR-205-3p and GLO1. RT-qPCR and Western-blot further verified that overexpression of miR-205-3p downregulated GLO1 expression levels.

GLO1 is present in all tissue, together with glyoxalase 2 and the cofactor glutathione, forms the glyoxalase system, the major detoxification enzyme system for carboxaldehyde, with increased expression reported in several tumours [25]. After verifying the targeting relationship between miR-205-3p and GLO1, we again performed rescue experiments and verified that GLO1 overexpression rescued the tumour suppressive effect of miR-205-3p on bladder cancer.Overall, miR-205-3p inhibits the biological behaviour of bladder cancer cells by negatively regulating GLO1.

MAPK is a widespread intracellular serine/threonine protein kinase that activated by mitogen, cytokines, neurotransmitters and other stimulis [26], thus converting extracellular signals into intracellular signals and exerting biological effects by regulating the expression and function of genes and proteins related to proliferation, apoptosis and autophagy [27]. To date, five parallel MAPK signalling pathways have been identified: extracellular signal-regulated protein kinase (ERK1/2), JNK/stress-activated protein kinase (SAPK), ERK5/big MAP kinase 1 (BMK1), p38MAPK (p38 mitogen-activated protein kinase), and ERK3/4 pathways [28]. ERK is mainly a transmitter of cell proliferation signals and activated by mitogen, while p38 is mainly activated by various extracellul ar stimuli, triggering a complex series of cellular stress transmissions [29]. MAPK has been reported in several studies to be associated with proliferation and apoptosis in bladder cancer [30–32]. Previous KEGG enrichment results from transcriptome sequencing suggest that miR-205-3p regulates the MAPK signalling pathway. At the same time, several studies have demonstrated that MAPK can interfere with the proliferation and apoptosis of BLCA cells. Therefore, we verified the changes in the phosphorylation level of P38/ERK after overexpression of miR-205-3p by molecular biology techniques and confirmed that overexpression of miR-205-3p in hibited the phosphorylation of P38/ERK in BLCA cells, while overexpression of GLO1 reversed this phenomenon. Furthermore, activation of P38/ERK reversed the effect of miR-205-3p and GLO1 on the proliferation and apoptosis of bladder cancer cells.

To further confirm the role of miR-205-3p in vivo,a xenograft mouse model was established.T24 cells stably expressing miR-205-3p as well as the corresponding negative control into nude mice to construct xenograft models.We found that the tumor volume and weight of LV-miR-205-3p group were lower than those of LV-NC group.Moreover,IHC analysis was carried out to detect the expression level of GLO1 and Ki-67 protein, which was consistent with the results in vitro,miR-205-3p inhibited the expression level of GLO1 and tumor proliferation.In addition, we also collected pathological tissues of bladder cancer patients, and divided them into high expression group of miR-205-3p and low expression group of miR-205-3p. It was found that the expression of GLO1 was down-regulated in the tissues with high expression of miR-205-3p, which was consistent with the cells experiment.

In summary, this research confirmed that miR-205-3p targeting inhibited GLO1 to mediated P38/ERK phosphorylation to suppress the biological behavior of bladder cancer cells.An in-depth study on the function of miR-205-3p may help to determine the therapeutic targets that are expected to improve the clinical prognosis of bladder cancer patients.

### Electronic supplementary material

Below is the link to the electronic supplementary material.

Supplementary Material 1

## Data Availability

The datasets generated and analyzed during the current study are available in the TCGA repository (https://portal.gdc.cancer.gov/).
