# Supplementary material for: MiR-205-3p suppresses bladder cancer progression via GLO1 mediated P38/ERK activation
Source: BMC Cancer. 2023 Oct 9;23:956. doi: 10.1186/s12885-023-11175-9 (PMC10563299; doi:10.1186/s12885-023-11175-9)

Fig3H GLO1

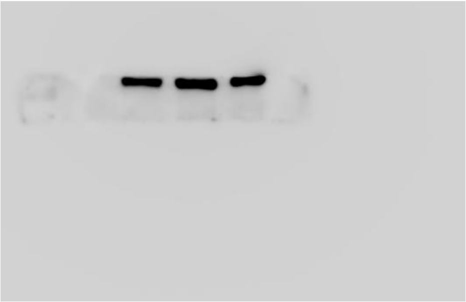

Fig3H β -actin

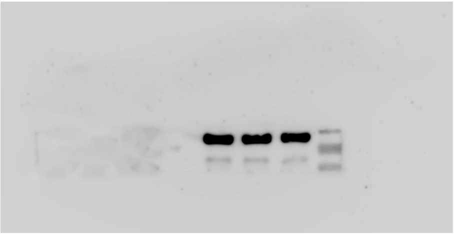

Fig4B

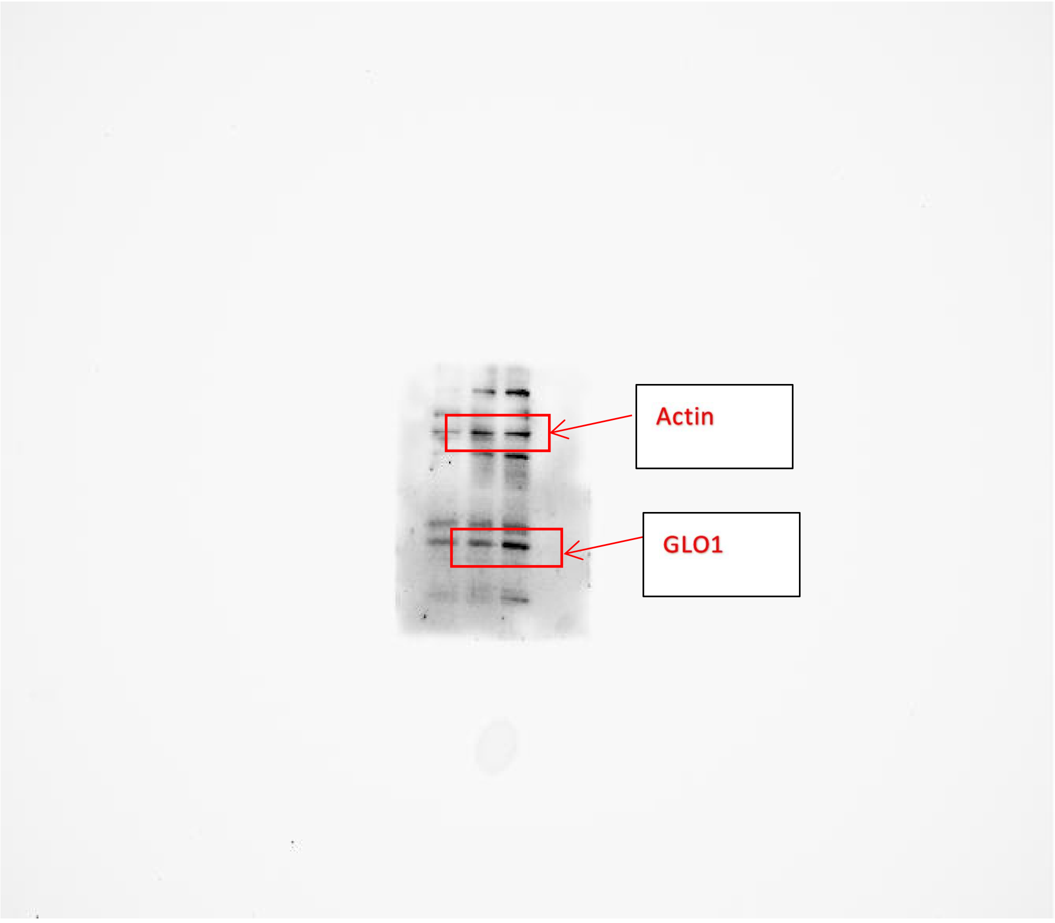

Fig-5 B E P38



Fig-5 B E p-P38

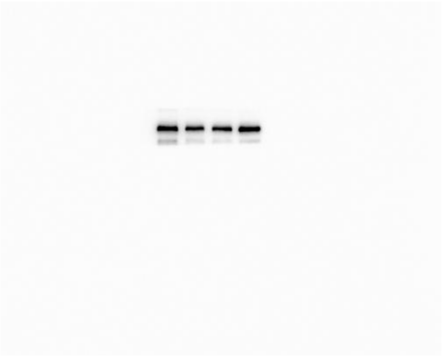

Fig-5 B E ERK

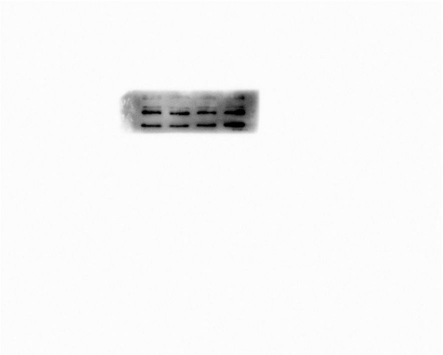

Fig-5 B E p-ERK

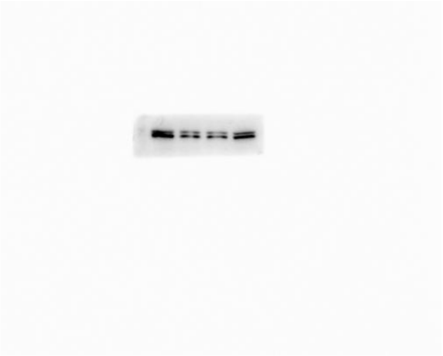

Fig-5 B E β -actin

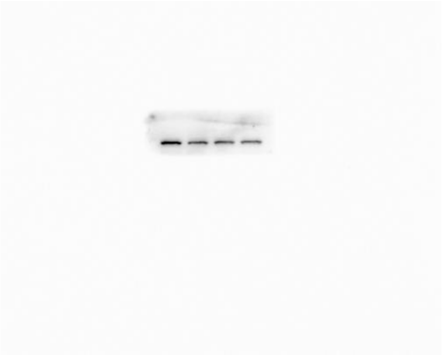

Fig-5 H P38



Fig-5 H p-P38

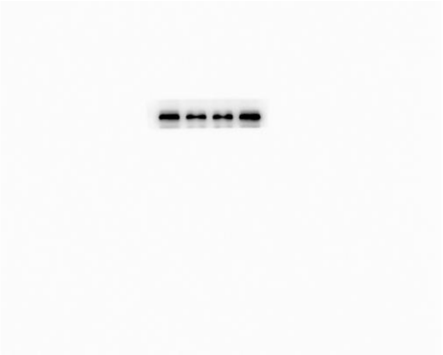

Fig-5 H ERK

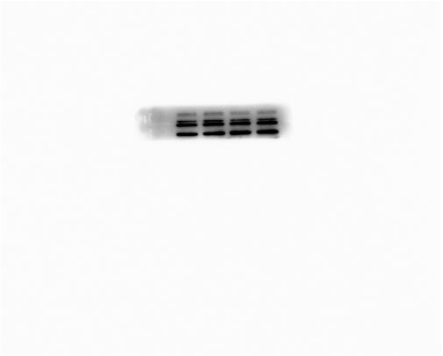

Fig-5 H p-ERK

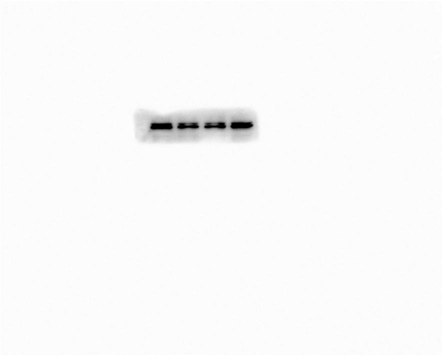

Fig-5 H β -actin

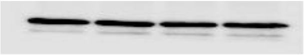

Supplement: Supplementary file 1 — Supplementary Material 1 [file 12885_2023_11175_MOESM1_ESM.pdf]
